# Hydrothermal vent fields discovered in the southern Gulf of California clarify role of habitat in augmenting regional diversity

**DOI:** 10.1098/rspb.2017.0817

**Published:** 2017-07-19

**Authors:** Shana K. Goffredi, Shannon Johnson, Verena Tunnicliffe, David Caress, David Clague, Elva Escobar, Lonny Lundsten, Jennifer B. Paduan, Greg Rouse, Diana L. Salcedo, Luis A. Soto, Ronald Spelz-Madero, Robert Zierenberg, Robert Vrijenhoek

**Affiliations:** 1 Department of Biology, Occidental College, Los Angeles, CA, USA; 2 Monterey Bay Aquarium Research Institute, Moss Landing, CA, USA; 3 School of Ocean Sciences, University of Victoria, Victoria, British Columbia, Canada; 4 Instituto de Ciencias del Mar y Limnología, Universidad Nacional Autónoma de México, Mexico City, Mexico; 5 Scripps Institution of Oceanography, La Jolla, CA, USA; 6 Department of Geology, Universidad Autónoma de Baja California, Mexico City, Mexico; 7 Earth and Planetary Sciences, University of California, Davis, Davis, CA, USA

**Keywords:** dispersal, community structure, foundation species, habitat suitability, faunal diversity, hydrothermal vents

## Abstract

Hydrothermal vent communities are distributed along mid-ocean spreading ridges as isolated patches. While distance is a key factor influencing connectivity among sites, habitat characteristics are also critical. The Pescadero Basin (PB) and Alarcón Rise (AR) vent fields, recently discovered in the southern Gulf of California, are bounded by previously known vent localities (e.g. Guaymas Basin and 21° N East Pacific Rise); yet, the newly discovered vents differ markedly in substrata and vent fluid attributes. Out of 116 macrofaunal species observed or collected, only three species are shared among all four vent fields, while 73 occur at only one locality. Foundation species at basalt-hosted sulfide chimneys on the AR differ from the functional equivalents inhabiting sediment-hosted carbonate chimneys in the PB, only 75 km away. The dominant species of symbiont-hosting tubeworms and clams, and peripheral suspension-feeding taxa, differ between the sites. Notably, the PB vents host a limited and specialized fauna in which 17 of 26 species are unknown at other regional vents and many are new species. Rare sightings and captured larvae of the ‘missing’ species revealed that dispersal limitation is not responsible for differences in community composition at the neighbouring vent localities. Instead, larval recruitment-limiting habitat suitability probably favours species differentially. As scenarios develop to design conservation strategies around mining of seafloor sulfide deposits, these results illustrate that models encompassing habitat characteristics are needed to predict metacommunity structure.

## 1. Introduction

Exploration of the seafloor continues to uncover new habitats fostering chemosynthetic communities in diverse tectonic settings. Altogether, 285 hydrothermal vent fields are presently confirmed, with hundreds more inferred and predicted (see https://www.interridge.org; [1,2]). Reduced volcanic and biogenic compounds dissolved in vent effluents (primarily H_2_S, CH_4_ and H_2_) support chemolitho-autotrophic microbial production as the primary nutrition for dense animal populations. As vent conditions are notably harsh with steep physico-chemical gradients, a highly specialized and habitat-restricted fauna has evolved [3,4], with many taxonomic similarities across ocean basins and tectonic backdrops (e.g. [5,6]). Nevertheless, contemporary connectivity and historical evolutionary relationships among vent taxa, and with those at other chemosynthetic habitats, are complex and remain incompletely understood [7,8].

Seafloor hydrothermalism can host large massive sulfide deposits that form as mineral-laden hot water emerges at the seafloor [9,10]. As interest grows in mining the metals in these deposits, so also does the need to understand possible consequences for the associated ecosystems [11] and how alteration and/or removal of vent fields will affect persistence of a regional vent assemblage spread among small habitat islands [12,13]. Thus far, connectivity models assume that vent site proximity is the primary driver of recruitment success [14]. Deep-ocean circulation tends to be constrained to axial valleys that course along mid-ocean ridge (MOR) systems, thereby reducing the loss of animal larvae by off-axis or cross-axis currents, and promoting long-distance connectivity among vent fields [15,16]; larval behaviour and local source populations also enhance survivorship [17,18]. Thus, MOR vent species are often distributed over large distances creating high similarity (low beta diversity) among assemblages (e.g. 9°50′ N to 21° N East Pacific Rise (EPR), 1200 km; [19]). However, within assemblage, alpha diversity is also affected by factors such as dispersal barriers, age of the vent, ecological succession, substratum type and vent fluid chemistry [20,21].

**Figure 1.**
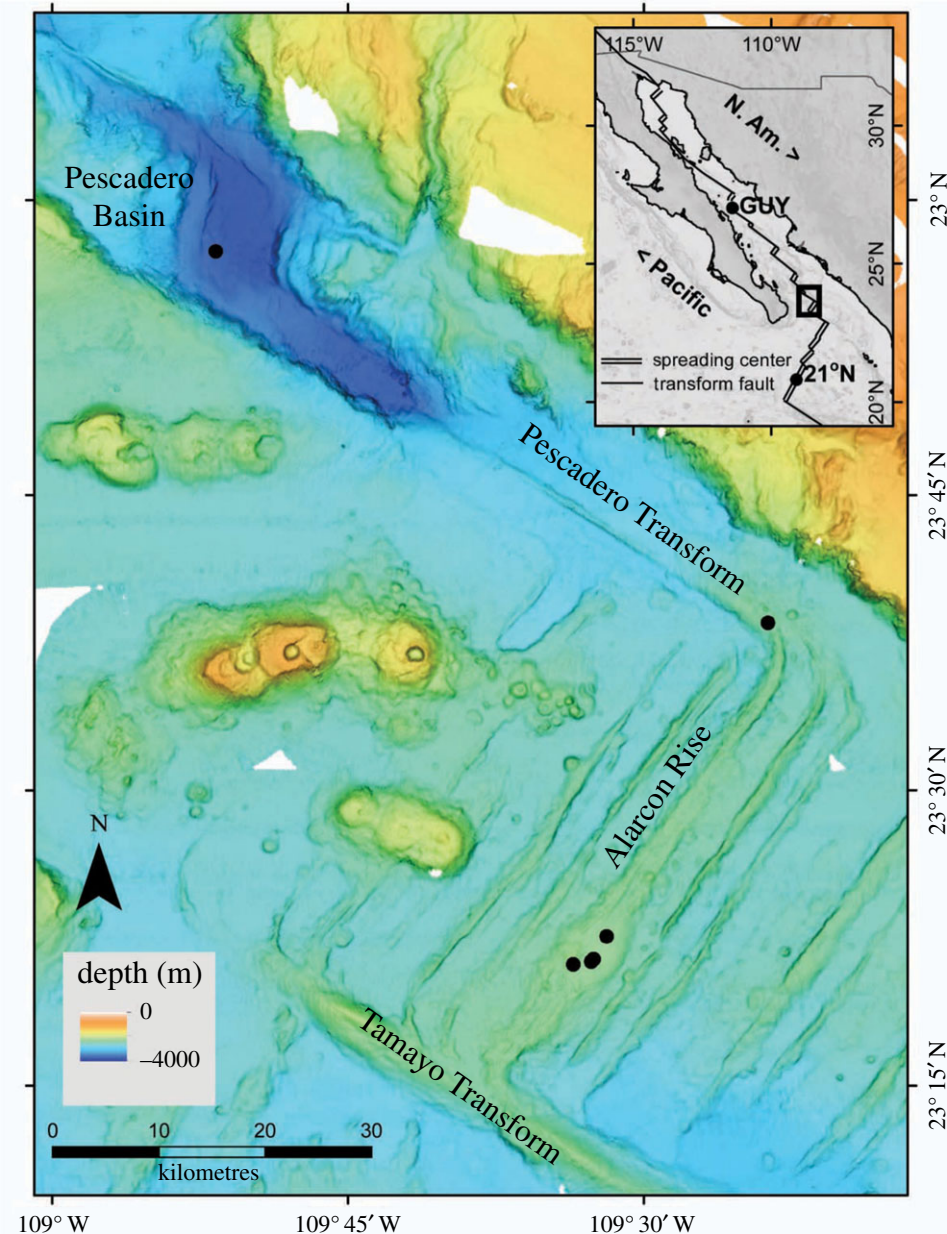
Bathymetric map of the southern Gulf of California showing the locations of the Pescadero Basin and Alarcón Rise vent fields, as well as the Pescadero Transform seeps (as black dots). Inset delineates the study area in the context of flanking vent fields in Guaymas Basin (GUY) and at 21° N East Pacific Rise (21° N).

The Gulf of California (GoC) formed as North America overrode the EPR, and now encompasses the northernmost segments of the EPR (figure 1). The Alarcón Rise (AR) is the northernmost segment of the EPR, before entering the GoC, with bare lava exposed. Further north in the GoC, deep extensional basins, typically filled with sediments, are separated by transform faults that accommodate shearing between the Pacific and North American plates. Here, high-temperature venting supports dense animal communities (e.g. Guaymas Basin; [22]). About 60 km north of the Guaymas vents, cold seeping fluids rich in hydrocarbons also host a chemosynthetic-based community [23]. Portail *et al*. [24,25] compared these vent and seep assemblages to find extensive taxonomic overlap (85% of species shared), and suggest that vents and seeps support a continuum of communities and of food web complexity. By extension, we expect fauna at any vent localities in the GoC also to have high overlap with each other and with the Guaymas Basin. The opportunity to test this hypothesis arose with the discovery of neighbouring vent fields in the southern GoC, when autonomous underwater vehicle (AUV) surveys and remotely operated vehicle (ROV) dives located vents in the Pescadero Basin (PB) [26,27] and on the AR [28]. Our study examines the composition of faunal assemblages at these two localities in comparison with vents at 21° N EPR and at approximately 27° N in Guaymas Basin, and implications for understanding community connectivity.

## 2. Material and methods

### (a) Study sites and biological sampling

High-resolution mapping surveys with the AUV *D. Allan B.* (Monterey Bay Aquarium Research Institute; MBARI) in 2012 and 2015 identified thermal anomalies and bottom features consistent with hydrothermal venting [28–30]. These data supported ROV (*Doc Ricketts*) dives to sample new vent fields and a new seep site (figure 1). The PB vent field near 24° N lies at 3700 m depth in a sedimented basin where a series of carbonate mounds and chimneys spread over an area of approximately 0.2 × 0.5 km [26]. The AR site, located 75 km south at 23.25° N, is a basalt-hosted system on the northernmost segment of the EPR. Venting is concentrated in three chimney fields along approximately 3 km of ridgecrest at 2300 m and in one field offset on an older flow at 2250 m. Between the PB and AR localities, seepage through sediments and outcrops occurs along the Pescadero transform fault (PTF), and supports chemosynthetic communities (at 23.64° N). During two expeditions, biological specimens were collected via ROV-mounted suction sampling and manual grabs using an articulating manipulator arm.

### (b) Morphological and molecular identification of fauna

For morphological analyses, specimens were preserved in 3.7% seawater-buffered formaldehyde. Identification was made to the lowest taxonomic level possible (electronic supplementary material, table S1). Representatives of all species, including holotypes for new species, have been deposited in the Benthic Invertebrate Collection at Scripps Institution of Oceanography. For molecular analyses, specimens were stored in chilled seawater until dissected or preserved by freezing in liquid nitrogen, at −80°C, or in 95% ethanol. Genomic DNA was extracted from specimens using the DNeasy^®^ Blood and Tissue kit (Qiagen, Inc., Valencia, CA, USA) according to the manufacturer's specifications. Molecular ‘barcoding’ was performed using PCR primers and loci based on each group (electronic supplementary material, table S2). Sequences were obtained on an ABI3700XL DNA sequencer with BigDye V3.1 (ThermoFisher, Waltham, MA, USA). Representative sequences for each species were submitted to GenBank (NCBI) with the following accession nos. KY581524-KY581549; KY684706-KY684728. Results were compared with sequences from GenBank using the BLASTn query, and from datasets of previously sequenced animals from the northern EPR and GoC. Taxonomic standards of the World Register of Marine Species (WoRMS) database were followed. Community membership among the four neighbouring vent fields was compared using Venny v. 2.1 [31]. Data from Portail *et al*. [24] were included for Guaymas Basin, as well as data from expeditions to the GoC and 21° N by the authors, with the ROV *Tiburon* and HOV *Alvin* (electronic supplementary material, table S1).

### (c) Larval detection

Seawater was sampled for biological particulate matter at the PB and AR hydrothermal vent fields, in and out of visible plumes, and over the PTF hydrocarbon seep. Roughly 60 l of water was collected using the ROV suction sampler operated at 20% power for 10 min from six different depths (up to approx. 50 m above chimneys). Most samples were acquired at a constant altitude from the seafloor (or top of chimney) as estimated by the ROV altimeter. Samples collected within the visible vent plume required the ROV to sometimes follow it as the current changed; however, these changes were relatively small and only varied by a few metres. Water was then filtered on 30–100 µm EMD Millipore™ nylon woven net filters, and prepared for Illumina high-throughput sequencing with a MiSeq sequencer (Illumina, Inc., San Diego, CA, USA) using the mitochondrial COI and ribosomal 28S primers indicated in the electronic supplementary material, table S2, with Illumina adaptors and barcodes [32]. Samples were sequenced at the Functional Genomics Facility, Stanford University, Palo Alto, CA, USA. Data were analysed in Qiime v. 1.9.1 [33] and Phyloseq, v. 1.19.1 [34] in the R package [35], within RStudio (v. 0.99.903). Taxonomic identity of DNA sequences was initially assigned by querying libraries constructed by all COI (+ COX1) or 28S sequences from GenBank, then via formatting for use within Qiime with the gb2qiime.py script (Mike McCann, 2014; see https://bitbucket.org/beroe/mbari-public/src/master/molecular/gb2qiime.py; electronic supplementary material, table S3).

### (d) Carbon and nitrogen isotope analysis

Specimens for measures of stable carbon and nitrogen isotopes were identified shipboard and frozen at −80°C. Tissues were extracted from large organisms, and in some cases, small individuals were pooled, to produce a minimum dry weight of 2 mg (dried at 60°C). Isotope determinations were made at the Stable Isotope Ratio Facility for Environmental Research, University of Utah. Values of *δ*^13^C are reported relative to Vienna Peedee Belemnite (VPDB) scale, and *δ*^15^N values are reported on the AIR scale.

### (e) Video transects

Video transects facilitated faunal type and abundance comparisons between PB and AR vents (transects covered 104 and 155 m^2^, respectively; table 1; electronic supplementary material, tables S4 and S5). Video was captured from an Ikegami HDL45 (1920 × 1080i) high-definition video camera. Transect width was determined using parallel 640 nm lasers positioned 29 cm apart with the ROV at a consistent distance (approx. 1 m) from seafloor or chimney. Transect lengths were calculated using coordinates within ArcGIS v. 10.3. Imagery was annotated using MBARI's Video Annotation and Reference System [36], yielding quantitative observations merged with additional data, such as depth and position. Animals greater than 1 cm in size were identified to the lowest possible taxon, aided by collected voucher specimens. As assignment to species was not always possible, morphologically distinct taxa were assigned an operational taxonomic unit (e.g. Actiniaria sp. 1; electronic supplementary material, table S1). Non-metric multi-dimensional scaling (NMDS) plots were calculated with MetaMDS (Bray–Curtis distances with a square root transformation and a Wisconsin double standardization (the default for NDMS in Vegan, v. 2.4-1)) of animal densities m^−2^ for each video transect with the R package [35], and illustrated with Ggplot2 [37] within RStudio (v. 0.99.903). The data were highly variable (many zero or very high values); therefore, Bray–Curtis distances of densities were normalized with a square root transformation and a Wisconsin double standardization, both of which are triggered in Vegan MetaMDS when values exceed default thresholds.

**Table 1.** Faunal type and abundance comparisons (no. m^−2^) between PB and AR vents via video transect data. (Symbol — denotes not observed in the video transects.)

	Pescadero Basin vents		Alarcón Rise vents	
taxa	average	max	average	max
Cnidaria				
Actiniaria sp. 1	23.2	67.9	0.4	2.0
Actiniaria sp. 2	—	—	9.5	131.4
Actiniaria—other	0.3	2.3	0.04	0.3
Cerianthidae	1.3	4.5	0.01	0.3
Zoanthidea	3.0	21.8	—	—
Annelida				
Alvinellidae	0.1	0.4	6.4	40.0
Amphinomidae	0.1	0.7	—	—
*Hesiolyra bergi*	—	—	0.1	0.8
*Nereis cf. sandersi*	—	—	0.2	1.1
*Oasisia* aff. *alvinae*	407.7	2423.0	28.9	200.0
*Peinaleopolynoe* sp. 1	15.6	42.8	0.1	0.7
*Riftia pachyptila*	—	—	139.2	730.1
*Protis* sp. 1	—	—	76.3	380.3
*Laminatubus* spp.	—	—	10.7	52.0
Serpulidae—other	0.01	0.1	1.7	15.2
Mollusca				
*Calyptogena magnifica*^a^	—	—	0.04	0.6
Vesicomyidae	0.01	0.1	0.01	0.1
Gastropoda	0.1	0.5	30.0	277.5
Patellogastropoda^b^	—	—	892.7	3822.6
Crustacea				
Amphipoda	0.03	0.3	30.0	409.4
*Bythograea thermydron*	—	—	2.9	11.2
Caridea	0.01	0.1	0.2	1.5
Galatheidae	0.1	0.3	1.7	8.0
Vertebrata				
*Thermarces cerberus*	0.1	0.4	9.4	61.2

^a^*Calyptogena magnifica*, which prefers hard substrate, is distinguished from the other vesicomyids, which typically inhabit soft sediments.

^b^Patellogastropods were so numerous at the Alarcón vents, they were distinguished from the other gastropods.

## 3. Results

### (a) Pescadero Basin vents

**Figure 2.**
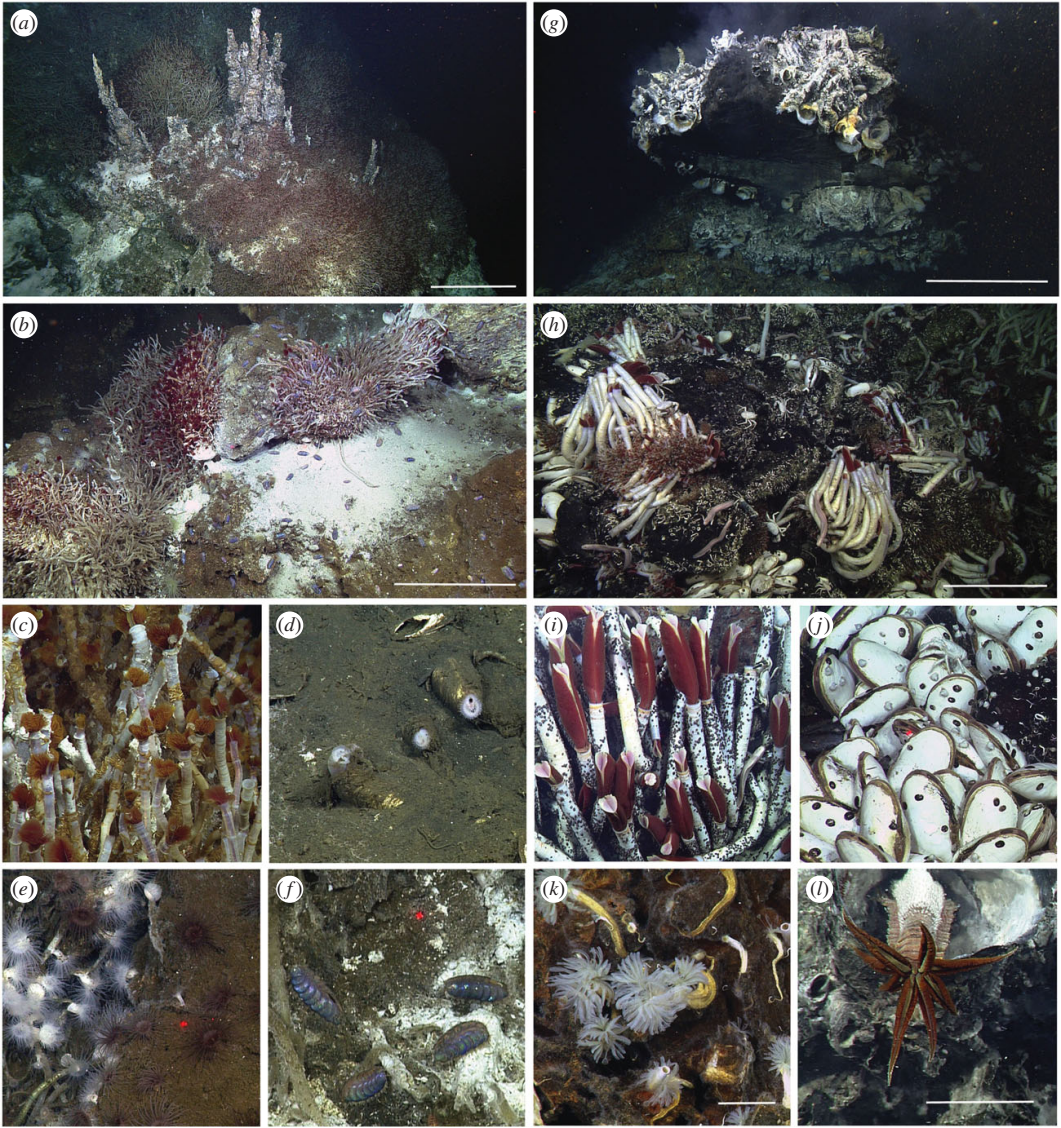
Images of venting structures and dominant animals from the PB vents (*a*–*f*) and AR vents (*g*–*l*). Carbonate mound with venting spires heavily colonized by tubeworms (*a*); closer view also shows polynoid scaleworms and anemones (*b*); *O. alvinae* tubes with yellow dorvilleid polychaetes (*c*); the clam *Archivesica* sp. 7 with white siphons emerging from sediments (*d*); a new actinarian species in two colour morphs (*e*); a new species of *Peinaleopolynoe* polynoid (*f*); diffusing high-temperature fluid from a flange bounded by tubes of polychaete *Al. pompejana* (*g*); serpulid polychaetes, ‘*Cal.*’ *magnifica* clams, recumbent *R. pachyptila* tubeworms colonize basalts with mobile foragers: bythograeid and galatheid crabs and zoarcid fish (*h*); close views of *R. pachyptila* (*i*) and *Cal. magnifica* (*j*) with limpet epifauna; a new species of *Protis* serpulid (*k*), *Al. pompejana* with dorsal microbial symbionts (*l*). Scale bars, (*a*–*c*, *g*–*h*) are 50 cm; scale bars (*k*–*l*) are 3 cm.

The PB vent field comprises three active hydrothermal mounds measuring 15–50 m base diameter, rising 12–25 m (figure 2*a*,*b*) with numerous active low mounds. Although vent fluids measured up to 290°C, there are no black smokers. Instead, these chimneys are composed of white and brown hydrothermal calcite. Fluids also emerge through surrounding rubble and sediments (at approx. 90°C). End-member fluids contained high concentrations of large aromatic hydrocarbons, hydrogen, methane and hydrogen sulfide (M. Lilley 2016, personal communication). pH values at approximately 6.5 (G. Elrod 2016, personal communication) are higher than any reported MOR vent, other than Lost City in the Atlantic [38,39].

Fauna consisted almost entirely of polychaetes. The siboglinid tubeworm *Oasisia* aff. *alvinae* overwhelmingly dominated (table 1 and figure 2*a*–*c*), while *Riftia pachyptila* was rare. *Oasisia* displayed two discrete morphotypes, akin to *Ridgeia* tubeworms at northeast-Pacific vents [40,41], but mitochondrial cytochrome *c* oxidase (mt*COI*) sequences were identical; within the *Oasisia* ‘group I’ clade of Hurtado *et al*. [42], and were greater than 99% similar to *Oasisia* from AR and EPR vents. A dorvilleid polychaete, *Ophryotrocha* cf. *akessoni*, was extremely abundant (figure 2*c*), in places covering the tubeworms entirely. Polynoid scaleworms included new species of *Peinaleopolynoe* (figure 2*f*), *Lepidonotopodium* and *Branchinotogluma,* as well as *Branchiplicatus cupreus*. Also abundant were the amphinomid *Archinome levinae* (described from methane seeps and Guaymas Basin; [43]) and *Nereis* cf. *sandersi*. The only alvinellids were *Paralvinella grasslei* and *Paralvinella* n. sp. (G. Rouse 2017, unpublished data).

Anemones occurred among the tubeworms, including one abundant species with white and red morphotypes (figure 2*e*; genetic sequencing of two mitochondrial loci did not differentiate between these two colour morphotypes). Scattered carbonates and sediments with emerging fluids featured other anemones, some burrowing, zoanthids and small beds of the vesicomyid clam, *Archivesica* sp. 7, known from methane seeps along the Costa Rica and Peru margins ([44,45]; figure 2*d*). Here, a newly described xenacoelomorph, *Xenoturbella profunda*, was collected [46]. Other taxa are indicated in the electronic supplementary material, table S1, including six first time sightings in the GoC.

Notably absent from PB vents were numerous polychaete species observed elsewhere in the GoC and EPR localities, especially ampharetids, hesionids, serpulids and the alvinellid genus *Alvinella* (electronic supplementary material, table S1). The PB fauna is further unusual in the low gastropod diversity and scarcity of decapod crustaceans (electronic supplementary material, table S1), such as bythograeid crabs. The galatheid crab *Munidopsis scotti* occurred in very low numbers.

The *δ*^13^C values of the PB vent fauna ranged from −37.9 to −13.1‰, with the most depleted values corresponding to two unidentified actinarians and the most enriched to the siboglinid tubeworms *R. pachyptila* and *Oasisia* aff. *alvinae* (figure 4; electronic supplementary material, table S6). The *δ*^15^N values ranged from −2.3‰ (*Archivesica* sp. 7) to 14‰ (Actiniaria group 7).

### (b) The Alarcón Rise vents

The AR vent field hosts four active vent sites with structures up to 33 m in height, including lateral flanges with hot water pooling below and multiple spires venting black particulate-laden effluent (figure 2*g*). Temperature maxima are up to 360°C; fluids have concentrations of hydrogen sulfide similar to other MOR sites, but a fraction of those measured at PB, and also little to no hydrogen, methane or hydrocarbons (M. Lilley 2016, personal communication). The bare rock of the AR segment is young basalt typical of mid-ocean spreading ridges. The chimneys formed by hydrothermal fluids are polymetallic sulfide deposits, also characteristic of such locations (figure 2*h*). All AR vent sites are similar in geochemistry and biology, and are treated here as a single locality.

While polychaetes again dominated the biomass on chimneys, there was greater taxonomic diversity at the AR vents. In vigorous venting, the alvinellids *Alvinella pompejana* (figure 2*g,l*) and *Alvinella caudata* formed dense colonies. The large hesionid *Hesiolyra bergi* was common among *Alvinella* tubes, as were other hesionids, including *Hesiospina vestimentifera* and three new *Hesiospina* species. Large *R. pachyptila* tubeworms covered moderately venting surfaces on chimneys and adjacent diffuse vents emanating from basalts (table 1 and figure 2*h*–*i*). The *Riftia* mt*COI* haplotypes were identical to those from Guaymas Basin through 32° S on the Pacific Antarctic Ridge [47]. In contrast with PB, *Oasisia* aff. *alvinae* was much less abundant (table 1). Alvinellids in the genus *Paralvinella* (*Paralvinella palmiformis* and *P. grasslei*) were present, along with the ampharetid *Amphisamytha fauchaldi*, known from the Guaymas Basin and methane seeps off Oregon and Costa Rica [48]. Nine species of scaleworm included three species each of *Branchinotogluma* and *Lepidonotopodium*. Many other species inhabited the *Riftia* tubes including very abundant limpets (*Lepetodrilus elevatus*, *Lepetodrilus cristatus* and *Euleptopsis vitrea*) and an undescribed species of *Peltospira* snail. Also common on AR chimneys were the predatory brachyuran crabs, *Bythograea thermodron* and *Cyanograea praedator*, and the zoarcid fish *Thermarces cerberus*.

On basalts around the chimneys, serpulid polychaetes were numerous, one species of which is known from vents further south (*Laminatubus alvini*; figure 2*k*), while two are new (*Laminatubus* and *Protis* n. spp.), but also found at Costa Rica methane seeps [49]. The galatheid squat lobsters, *Munidopsis recta* and *Munidopsis lentigo*, were frequently observed*.* Dense clusters of the giant clam, ‘*Calyptogena*’ *magnifica* (figure 2*j*), occupied venting cracks in basalts (unresolved genus assignment; [45]). Other taxa are indicated in the electronic supplementary material, table S1, including 23 range extensions of known species. Notably absent from AR vents were numerous other limpets (e.g. *Neolepetopsis, Hirtopelta*) and snails (e.g. *Provanna*) observed elsewhere in the GoC and EPR localities.

Stable isotopes were most depleted in the bivalve *Cal. magnifica* (*δ*^13^C at −34.2‰; *δ*^15^N at −4.6‰). The polychaetes *Al. pompejana* and *R. pachyptila*, hosts of external and internal sulfur-oxidizing symbionts, respectively, had enriched *δ*^13^C signatures of −9.4 and 11.1‰ (figure 4). The most enriched *δ*^15^N value corresponded to the distal vent-associated sponge *Caulophacus cyanae* (17.6‰).

### (c) The Pescadero transform fault seeps

The 2400 m deep hydrocarbon seep on the PTF lies at 23°38.5′ N/108°23.6′ W. While most of the uplifted sediment hills were surrounded by younger lava flows (e.g. [50]), only discrete low-temperature seepage was observed along the transform in volcanic rubble and sediments. The seeps were dominated by scattered dense patches of siboglinid tubeworms, *Escarpia spicata* and *Lamellibrachia barhami* (electronic supplementary material, figure S2). Abundant polychaetes included the amphinomid *Ar. levinae*, the serpulid species, *Laminatubus* sp. (shared with the AR vents) and a putatively new species of *Branchinotogluma* polynoid. The vesicomyid clams *Calyptogena costaricana* and *Archivesica.* mt-V [44] were common. Several gastropod genera more typically associated with hydrothermal vents were observed, including *Provanna ios, Paralepetopsis* sp. and *Neolepetopsis* aff. *gordensis*. *Munidopsis* squat lobsters, which are typically abundant at eastern Pacific vents and seeps, were not observed. The *δ*^13^C signatures of *L. barhami* and *Es. spicata* were slightly more depleted than those of siboglinids from the PB and AR vents (electronic supplementary material, table S6), while the single *Calyptogena* specimen was notably more depleted in both isotopes than *Cal. magnifica* at AR (figure 4).

### (d) Taxonomic overlap among Gulf of California and East Pacific Rise vent fields

The closest known hot vents in the GoC are at Guaymas Basin 425 km to the north of PB and 21° N EPR 285 km to the south of AR (figure 1 inset). In all, 116 macrofaunal taxa (greater than 1 cm size) were recognized as distinct species from the four main vent fields, many of which are undescribed (electronic supplementary material, table S1). The list is undoubtedly incomplete as approximately 48 h of ROV bottom time limited sampling, and inconspicuous animals may not be recognized in video transects. Of the species recovered in our study, only three taxa (*R. pachyptila, P. grasslei* and *Nereis* cf*. sandersi*) occur at all four locations (electronic supplementary material, table S1). Two additional species (*Thermarces cerberus* and *Oasisia* aff*. alvinae*) are shared among the southern localities (21° N EPR, Alarcón and Pescadero), but not with Guaymas Basin vents (electronic supplementary material, table S1).

The PB vents host a limited and specialized fauna in which 17 of 26 species are unknown at other regional vents and many are new species (electronic supplementary material, table S1). This sedimented site shares six species with Guaymas (also sedimented) to the north. Despite their 75 km proximity, the PB and AR vent fields share only seven of the 61 taxa observed (electronic supplementary material, table S1). While AR also has five species in common with Guaymas Basin vents, its fauna is more similar to basalt-hosted vent communities along the northern EPR, including 21° N EPR with which it shares 67% of its fauna (28 out of 42; electronic supplementary material, table S1). Here, ‘exclusivity’ is lower, but at least eight of 42 species are new to science. At the level of family, notable differences also appear: while AR shares 19 out of 23 families with the distant 21° N EPR site, there are only 12 in common with the more proximal PB locale. Despite the sedimented setting, PB shares only nine of 16 families with the Guaymas Basin vents.

### (e) Faunal abundance comparisons between vent fields

**Figure 3.**
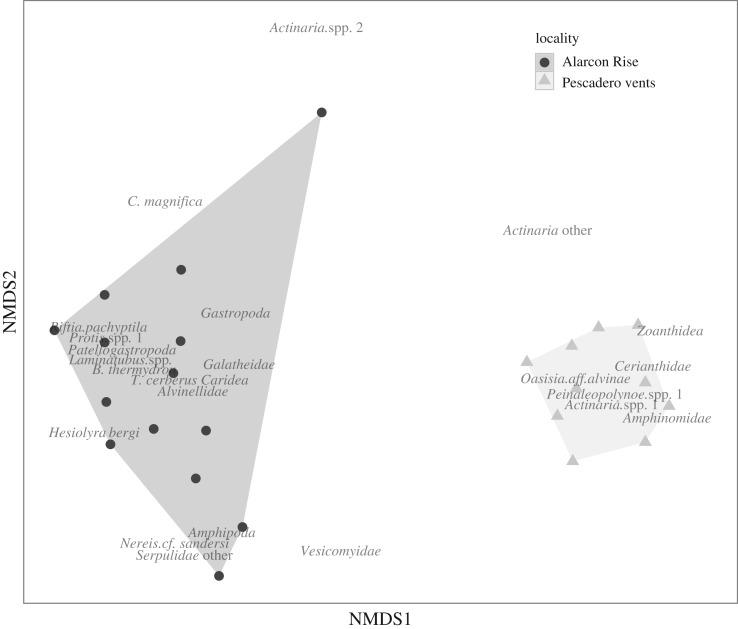
NMDS plot of animal densities m^−2^ for video transects from both locations; PB vents, AR vents (calculated via Bray–Curtis distances with a square root transformation and a Wisconsin double standardization). Triangles and the light grey polygon represent transect replicates at four PB vent chimneys (1–4 replicates per vent field), and circles and the dark grey polygon represent transect replicates from three AR vent chimneys (four to five replicates per field). Influential taxa are shown in text. The overall stress is 0.113. The non-metric fit *R*^2^ is 0.987 and the linear fit *R*^2^=0.951 for the ordination distance versus the observed dissimilarity.

Video transects of active chimneys revealed markedly different patterns of macrofaunal abundance between PB and AR (table 1; electronic supplementary material, tables S4 and S5). NMDS comparison of animal densities showed complete separation of the two localities, with observed community membership from all 10 PB transects clustered closely together, to the exclusion of the 14, more dispersed, AR transects (figure 3). A Shepard plot of NMDS scores revealed very low stress (less than 0.12) for a two-dimensional plot (data not shown). At PB vents, *Oasisia* aff*. alvinae* tubeworms enveloped the large chimneys and mounds in densities up to 2400 individuals m^−2^ (avg. = 408; figure 2*a*,*b*). At AR vents, *Oasisia* density was much lower and large groups were infrequent. By contrast, giant *R. pachyptila* tubeworms dominated AR vents to a maximum of 730 individuals m^−2^ (figure 2*h*,*i*), but were relatively scarce at PB vents (table 1). On the seafloor at both AR and PB, vesicomyid clam abundances are fairly similar, although species differ (table 1; electronic supplementary material, table S1). The *Alvinella* species at AR are notable in their contribution to biomass in intense fluid flows on chimneys.

Among the grazers and small predators on chimneys at PB, the relative contribution of polynoids such as *Peinaleopolynoe* n. sp. was high (up to 27 individuals m^−2^; figure 2*f*), while at AR gastropods, such as the patellogastropod *Le. elevatus* (at over approx. 3800 individuals m^−2^; figure 2*i*,*j*), and amphipods (up to 400 m^−2^) were high. Larger roving predators were much more abundant at AR (table 1), while nereids, crabs and fishes were mostly absent at PB. Cnidarian suspension feeders on the vent periphery were present at both localities, although species differed. At AR, but not PB, dense fields of serpulids (to 380 m^−2^; figure 2*k*) colonized the basalts and high numbers of galatheid crabs, presumably *M. lentigo* and *M. recta*, ranged far from the vents (to 8 m^−2^; table 1).

### (f) Detection of larval DNA

Illumina sequencing of COI and 28S rRNA amplicons in water filtered above PB and AR vent fields revealed 12 taxa associated with reducing environments (electronic supplementary material, table S3). AR water contained signatures from resident annelids (*R. pachyptila, Oasisia alvinae* and *Al. pompejana*) and molluscs (*‘Cal.’ magnifica, Le. elevatus* and *Provanna* n. sp.)*.* By contrast, the only larval species detected in the PB vent samples were non-residents, including the annelid *Nicomache* sp., the hexacoral *Maractis* sp. and the clam *C. costaricana*. DNA from *C. costaricana* clams was detected from all three southern Gulf localities, but adults were only verified from the PTF seep. The PTF water samples also contained larval DNA from several species associated with the PB and/or AR vents, that did not occur as adults at PTF: *R. pachyptila* ‘*Cal.*’ *magnifica, L. elevatus*, *Melanodrymia aurantiaca* and the shrimp *Lebbeus carinatus* (electronic supplementary material, table S3).

## 4. Discussion

Notably different faunal communities colonize the two newly discovered neighbouring localities; only seven of 61 species are shared and 10 additional species are new to science, despite proximity to known vents. Computational models suggest that larval lifespans, distances between localities and ocean currents strongly influence the composition of vent communities in the western Pacific [51]. Geostrophic estimates show distinct two-way flow near bottom between PB and AR with a Pacific-ward flow to the east and inflows to the west [52]; thus, two-way larval exchange is possible, and the 75 km distance between PB and AR vent fields lies within the average dispersal distances for most worm, clam and crustacean larvae [18,53,54]. While the recent Lagrangian connectivity model of Montaño-Cortés *et al.* [55] does not encompass the AR field, it illustrates particles crossing basin boundaries with some vertical component in four- and eight-week runs. Thus, the distinction of macrofauna between PB and AR locales suggests that community composition is related less to geographical proximity and larval supply than to habitat suitability. Biological interactions (e.g. priority effects, competitive interactions) and stochasticity in colonization can also strongly influence community structure [56,57]; however, the large differences between the PB and AR vent communities probably exceed the scale expected for these factors. Indeed, the size and extent of the deposits at both localities indicate a long-term stability in hydrothermalism sustaining community development; smaller Guaymas chimneys are around 4000 years old [58]. The approximately 1300 m depth differences may play a role in differentiating the fauna [59–61] because of hydrostatic pressure effects on animals [62], but the dominant PB vent species were not novel or unknown from other regional vents. Depth transcendence does occur; e.g. in the Mariana Backarc Basin, the same vent species occur from 1500 to 3600 m over a distance of 600 km [63]. In the absence of obvious biogeographic barriers, we hypothesize that other physical and chemical parameters control the structure of PB faunal communities.

PB and AR vent fields differ in underlying substratum composition, with carbonate structures and rubble embedded in sediment versus sulfide chimneys and mounds on basalt, respectively. The work of Portail *et al*. [24], also in the GoC, determined that macrofaunal community composition was significantly influenced by substratum type, rather than fluid temperature or pH. Deep sediments at the PB vents probably limit the presence of ‘*Cal.*’ *magnifica*, serpulids and some anemones, all of which inhabit basalts or other hard substrata. Similarly, the paucity of gastropods, and possibly polynoids, may relate to limited substratum availability.

In their comparative study of six seep and four vent fields within a 60 km range in Guaymas Basin, Portail *et al*. [24] found high faunal similarity among localities; all vent families occurred at the seeps. However, at least seven of 16 animal families from PB are absent at Guaymas seeps or vents, revealing low similarity between these sedimented sites. Considering the additional dissimilarity between the PB and AR vents to the south (15 families here are not shared between locales), we suggest their hypothesis of ‘continuity among deep-sea seep and vent ecosystems’ ([24], p. 5455) may not apply universally. The present study suggests that more substantial diversification and differentiation can result from variation in physical and chemical factors.

The PB fluids have notably elevated levels of H_2_, CH_4_ and large hydrocarbons (M. Lilley 2016, personal communication), and pH. Fluids here, like the Guaymas Basin vents, emerge through thick sediments where hydrothermal alteration of sedimentary organic matter produces methane and hydrocarbons [64], resulting in conditions that differ greatly from the original end-member fluids [65]. Concentrations of reduced compounds, particularly methane, are hypothesized to influence variability in macrofaunal composition [24,66]. How these reduced gases directly affect the macrofauna is not known; however, biochemical alteration of vent fluids, including the accumulation of both hydrogen and methane, has implications for the structure and functioning of both the free-living microbial communities, as well as bacterial symbionts hosted by foundation fauna.

**Figure 4.**
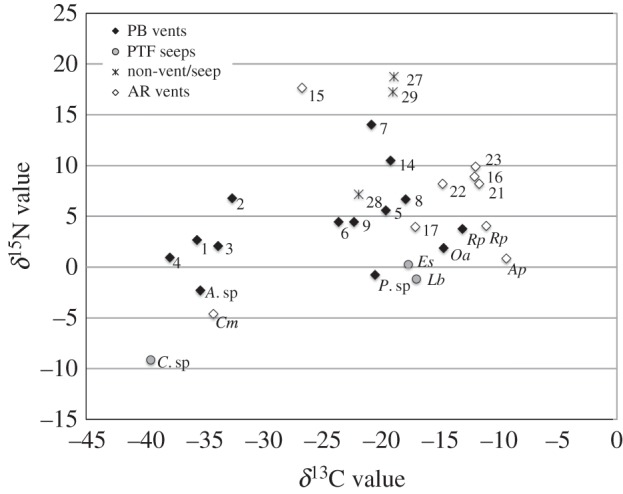
Biplots of *δ*^13^C and *δ*^15^N values of the fauna from the PB vents, AR vents and PTF seeps. Animals with symbionts are indicated by their genus and species abbreviation (ex. Rp*, Riftia pachyptila*), while all others are noted by numbers corresponding to the electronic supplementary material, table S6.

Stable isotope results suggest that organic carbon sources may differ between AR and PB vent fields (figure 4). In general, the *δ*^13^C values of tested animals were more depleted at PB (from −37.9 to −13.1‰), compared with AR (−34.2 to −9.4‰). The most depleted value was recorded from *C. costaricana* from PTF (−39.5‰; electronic supplementary material, table S6). This implies that some of the dissolved inorganic carbon has a methane origin, perhaps a CO_2_ intermediate. The highly depleted *δ*^13^C values for some of the larger anemones also reveal this influence.

The deep carbonate-hosted vents at PB form a novel environment with a high alpha diversity in the spatially restricted area of the southern GoC; a majority of species are not recorded from elsewhere in the GoC. Moreover, the high habitat variability among vent localities from north EPR through the GoC promotes high beta diversity among communities over a relatively short distance. Larval DNA detection of taxa not present as adults suggests that dispersal among the southern GoC localities is possible. However, the PB site is biologically distinct from other regional vents; foundation species of tubeworms and clams differ, and major space colonists (i.e. serpulids, patellogastropods) are absent. Therefore, structuring factors for faunal composition and food-webs must include geological setting, vent fluid characteristics and substratum heterogeneity, all of which affect habitat suitability and limit recruitment of select species.

High variability of fauna and habitat conditions among spatially discreet communities creates challenges for managing conservation approaches in the ocean. Deep-sea mining of polymetallic sulfides will target large deposits that may support vent communities [12,67]. Managing consequences of such extraction includes the need to better understand connectivity patterns and conservation of regional deep-sea vent faunal networks (e.g. [51,68]). However, the framework to support management and conservation must include assessment of the habitat characteristics that determine current community composition, especially in the context of ‘recovery’ at a mine site that has undergone drastic habitat alteration and loss of species. The vent fields of the southern GoC provide an excellent opportunity to explore how habitat diversity influences biodiversity distribution.

## Supplementary Material

Supplemental Materials - Total

## Data accessibility

This article has no additional data.
