# Supplementary material for: Hydrothermal vent fields discovered in the southern Gulf of California clarify role of habitat in augmenting regional diversity
Source: Proc Biol Sci. 2017 Jul 19;284(1859):20170817. doi: 10.1098/rspb.2017.0817 (PMC5543219; doi:10.1098/rspb.2017.0817)
Supplement: Supplemental Materials - Total [file rspb20170817supp1.pdf]

**SUPPLEMENTAL FIGURES and TABLES**

Figure S1: Map of each vent field showing locations of multiple hydrothermal structures and approximate video transect locations. Note the tilted view on the PB inset featuring active chimneys in the north.

Figure S2: The Pescadero Transform Fault seep was dominated by dense aggregations of the siboglinid tubeworms (A) *Escarpia spicata* and (B) *Lamellibrachia barhami*.

Table S1: Taxa presence and absence at five sites, based on past and current collections, video transects, and literature. Pescadero Basin and Alarcón Rise vent fields, and the closest known hot vents to the north and south (Guaymas Basin and 21°N EPR). Pescadero Transform Fault seeps.

Table S2: PCR primers and conditions used to amplify and sequence genes for identification for each taxonomic group extracted.

Table S3: Larval detection, via environmental DNA analysis from 100-µm filtered seawater collected at 5 different depths at each site. Taxa detected in the water column are indicated via an *l.* Taxa collected or observed via video are indicated by an x.

Table S4: Faunal type and abundance (# $m^{-2}$) for Pescadero Basin vents, identified from 10 video transects at four venting structures.

Table S5: Faunal type and abundance (# $m^{-2}$) for Alarcón Rise vents, identified from 14 video transects at three vent fields.

Table S6: Mean (±SD) and individual values of $\delta^{13}C$ and $\delta^{15}N$ of the fauna from Pescadero Basin (PB), Alarcón Rise (AR), and Pescadero Transform Fault (PTF). N = number of individuals.

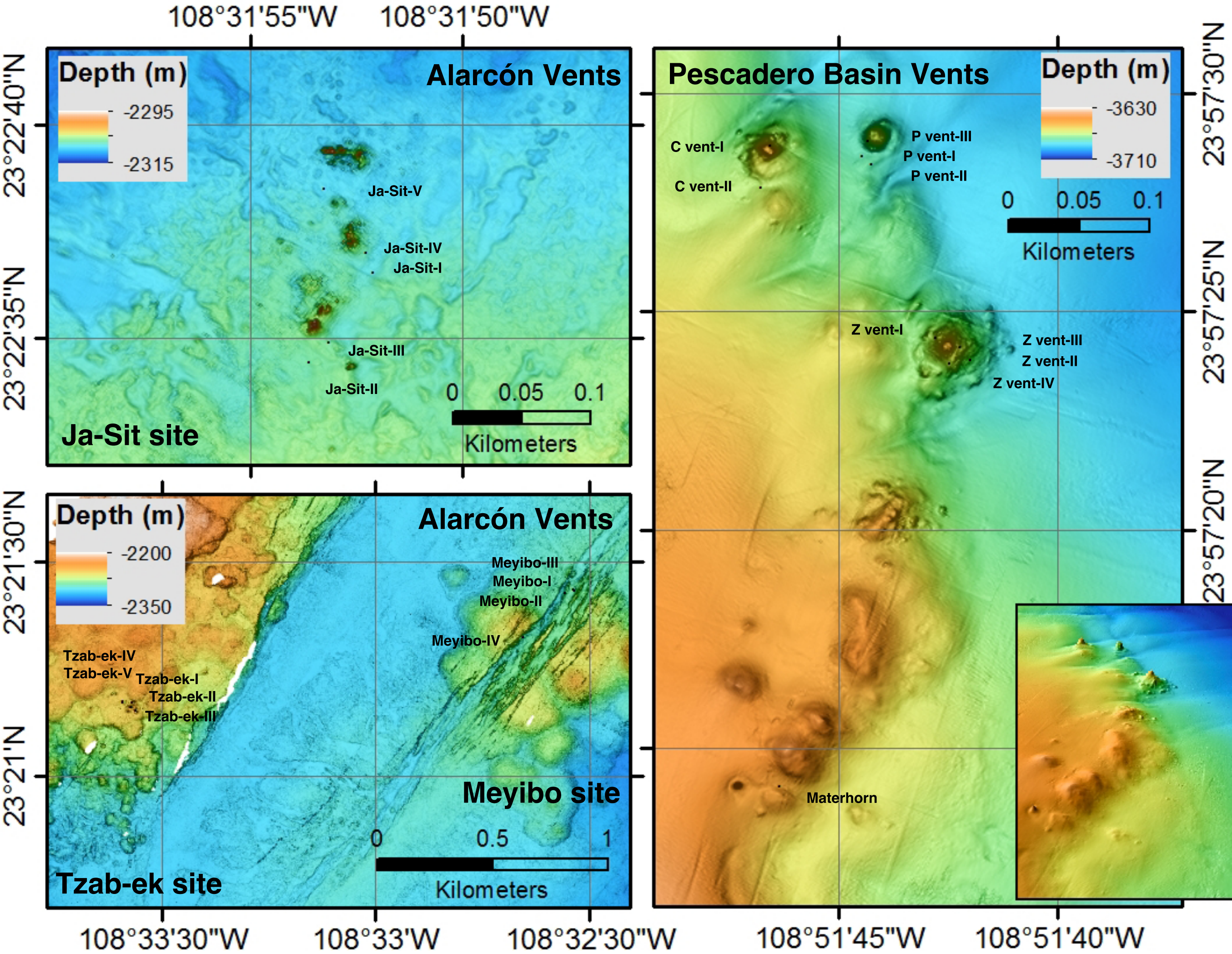
108°31'55"W
108°31'50"W
Depth (m)
-2295
-2315
Alarcón Vents
Ja-Sit-V
Ja-Sit-IV
Ja-Sit-I
Ja-Sit-III
Ja-Sit-II
0 0.05 0.1
Kilometers
Ja-Sit site
23°22'40"N
23°22'35"N
Pescadero Basin Vents
Depth (m)
-3630
-3710
C vent-I
C vent-II
P vent-III
P vent-I
P vent-II
0 0.05 0.1
Kilometers
Z vent-I
Z vent-III
Z vent-II
Z vent-IV
Materhorn
23°57'30"N
23°57'25"N
23°57'20"N
Depth (m)
-2200
-2350
Alarcón Vents
Meyibo-III
Meyibo-I
Meyibo-II
Meyibo-IV
Tzab-ek-IV
Tzab-ek-V
Tzab-ek-I
Tzab-ek-II
Tzab-ek-III
Meyibo site
0 0.5 1
Kilometers
Tzab-ek site
23°21'30"N
23°21'N
108°33'30"W
108°33'W
108°32'30"W
108°51'45"W
108°51'40"W

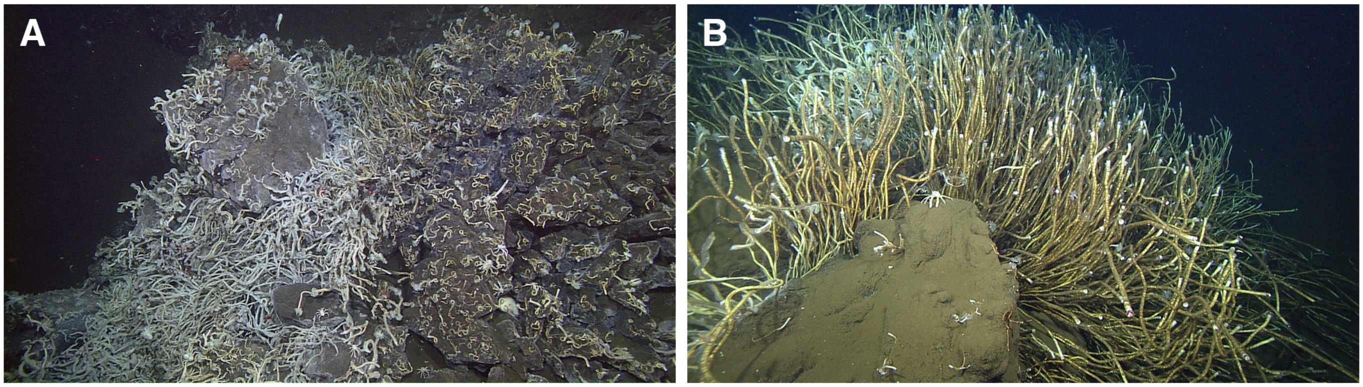
A
B

Supplemental Table 1: Taxa presence and absence at five sites, based on past and current collections, video transects, and literature.
Pescadero Basin and Alarcón Rise vent fields, and the closest known hot vents to the north and south (Guaymas Basin and 21°N EPR).

| Phylum | Family | Taxon | 21N EPR | AR vents | PB vents | PTF Seeps | GUY vents |
|---|---|---|---|---|---|---|---|
| Annelida | Alvinellidae | *Alvinella caudata* | 1 | **1** | 0 | 0 | 0 |
| Annelida | Alvinellidae | *Alvinella pompejana* | 1 | **1** | 0 | 0 | 0 |
| Annelida | Alvinellidae | *Paralvinella grasslei* | 1 | 1 | 1 | 0 | 1 |
| Annelida | Alvinellidae | *Paralvinella n. sp.* | 0 | 0 | 1 | 0 | 0 |
| Annelida | Alvinellidae | *Paralvinella palmiformis* | 1 | **1** | 0 | 0 | 0 |
| Annelida | Alvinellidae | *Paravinella bacteriocola* | 0 | 0 | 0 | 0 | 1 |
| Annelida | Ampharetidae | *Amphisamytha galapagensis* | 1 | 0 | 0 | 0 | 0 |
| Annelida | Ampharetidae | *Amphisamytha fauchaldi* | 0 | 1 | 0 | 0 | 1 |
| Annelida | Amphinomidae | *Amphinome sp.* | 0 | 0 | 0 | 0 | 1 |
| Annelida | Amphinomidae | Amphinomidae sp. 1 | 0 | 0 | 1 | 0 | 0 |
| Annelida | Amphinomidae | *Archinome levinae* | 0 | 0 | 1 | 1 | 1 |
| Annelida | Cirratulidae | *Aphelochaeta sp.* | 0 | 0 | 0 | 0 | 1 |
| Annelida | Dorvilleidae | *Exallopus jumarsi* | 0 | 0 | 0 | 0 | 1 |
| Annelida | Dorvilleidae | *Ophryotrocha cf. akessoni 2* | 0 | 1 | 0 | 0 | 0 |
| Annelida | Dorvilleidae | *Ophryotrocha cf. akessoni1* | 0 | 0 | 1 | 0 | 0 |
| Annelida | Dorvilleidae | *Ophryotrocha platykephale* | 0 | 0 | 0 | 0 | 1 |
| Annelida | Dorvilleidae | *Parougia sp.* | 0 | 0 | 0 | 0 | 1 |
| Annelida | Hesionidae | *Hesiolyra bergi* | 1 | **1** | 0 | 0 | 0 |
| Annelida | Hesionidae | *Hesiospina cf. vestimentifera1* | 1 | **1** | 0 | 0 | 0 |
| Annelida | Hesionidae | *Hesiospina cf. vestimentifera2* | 0 | 1 | 0 | 0 | 0 |
| Annelida | Hesionidae | *Hesiospina cf. vestimentifera3* | 0 | 1 | 0 | 0 | 0 |
| Annelida | Hesionidae | *Hesiospina cf. vestimentifera4* | 0 | 1 | 0 | 0 | 0 |
| Annelida | Hesionidae | *Nereimyra alvinae* | 0 | 0 | 0 | 0 | 1 |
| Annelida | Hesionidae | *Sirsoe grasslei* | 0 | 0 | 0 | 0 | 1 |
| Annelida | Maldanidae | *Nicomache cf. arwidssoni* | 1 | **1** | 0 | 0 | 0 |
| Annelida | Nereididae | *Nereis cf. sandersi* | 1 | 1 | 1 | 0 | 1 |
| Annelida | Pilargidae | *Sigambra sp.* | 1 | 0 | 0 | 0 | 0 |
| Annelida | Polynoidae | *Bathykurila guaymasensis* | 0 | 0 | 0 | 0 | 1 |
| Annelida | Polynoidae | *Branchinotogluma hessleri* | 1 | **1** | 0 | 0 | 0 |
| Annelida | Polynoidae | *Branchinotogluma n.sp. 1* | 0 | 1 | 0 | 0 | 0 |
| Annelida | Polynoidae | *Branchinotogluma n.sp. 2* | 0 | 1 | 0 | 0 | 0 |
| Annelida | Polynoidae | *Branchinotogluma n.sp. 3* | 0 | 0 | 0 | 1 | 1 |
| Annelida | Polynoidae | *Branchinotogluma n.sp. 4* | 0 | 0 | 1 | 0 | 0 |
| Annelida | Polynoidae | *Branchinotogluma sandersi* | 1 | 1 | 0 | 0 | 1 |
| Annelida | Polynoidae | *Branchiplicatus cupreus* | 1 | 0 | 1 | 0 | 1 |
| Annelida | Polynoidae | *Iphionella risensis* | 1 | **1** | 0 | 0 | 0 |
| Annelida | Polynoidae | *Lepidonotopodium fimbriatum* | 1 | **1** | 0 | 0 | 0 |
| Annelida | Polynoidae | *Lepidonotopodium n sp.* | 0 | 1 | 1 | 0 | 0 |
| Annelida | Polynoidae | *Lepidonotopodium williamsae* | 1 | **1** | 0 | 0 | 0 |
| Annelida | Polynoidae | *Macellicephalinae sp. 1* | 0 | 0 | 0 | 1 | 0 |
| Annelida | Polynoidae | *Macellicephalinae sp. 2* | 0 | 0 | 1 | 0 | 0 |
| Annelida | Polynoidae | *Peinaleopolynoe n. sp.1* | 0 | 0 | 1 | 1 | 0 |
| Annelida | Polynoidae | *Thermiphione tufari* | 1 | 0 | 0 | 0 | 0 |
| Annelida | Serpulidae | *Laminatubus alvini* | 1 | **1** | 0 | 0 | 0 |
| Annelida | Serpulidae | *Laminatubus n. sp.* | 0 | 0 | 0 | 1 | 1 |
| Annelida | Serpulidae | *Protis hydrothermica* | 1 | 0 | 0 | 0 | 0 |
| Annelida | Serpulidae | *Protis n. sp.* | 0 | 1 | 0 | 0 | 0 |
| Annelida | Siboglinidae | *Escarpia spicata* | 0 | 0 | 0 | 1 | 1 |
| Annelida | Siboglinidae | *Lamellibrachia barhami* | 0 | 0 | 0 | 1 | 0 |
| Annelida | Siboglinidae | *Oasisia aff. alvinae (hap I)* | 1 | **1** | 1* | 0 | 0 |
| Annelida | Siboglinidae | *Riftia pachyptila* | 1 | 1 | 1 | 0 | 1 |
| Annelida | Spionidae | *Lindaspio dibranchiata* | 0 | 0 | 0 | 0 | 1 |
| Annelida | Spionidae | *Prionospio sandersi* | 0 | **1** | 0 | 0 | 0 |
| Chordata | Zoarcidae | *Thermarces cerberus* | 1 | 1 | 1 | 0 | 0 |
| Cnidaria | Actiniaria | Actinaria sp. 1 | 0 | 0 | 1 | 0 | 0 |
| Cnidaria | Actiniaria | Actinaria sp. 2 | 0 | 1 | 1 | 0 | 0 |
| Cnidaria | Actiniaria | *Zoantharia* | 0 | 0 | 1 | 0 | 0 |
| Arthropoda | Amphipoda | Amphipod sp. 1 | 0 | 0 | 1 | 0 | 0 |
| Arthropoda | Amphipoda | *Ventiella sulfuris* | 1 | **1** | 0 | 0 | 0 |
| Arthropoda | Bythograeidae | *Bythograea thermydron* | 1 | **1** | 0 | 0 | 0 |
| Arthropoda | Bythograeidae | *Cyanogrea praedator* | 1 | 0 | 0 | 0 | 0 |
| Arthropoda | Eolepadidae | *Neolepas zevinae* | 1 | **1** | 0 | 0 | 0 |
| Arthropoda | Galatheidae | *Munidopsis lentigo* | 1 | **1** | 0 | 0 | 0 |
| Arthropoda | Galatheidae | *Munidopsis recta* | 1 | **1** | 0 | 0 | 0 |
| Arthropoda | Galatheidae | *Munidopsis scotti* | 0 | 0 | 1* | 0 | 0 |
| Arthropoda | Hippolytidae | *Lebbeus carinatus* | 0 | 1 | 0 | 0 | 0 |
| Mollusca | Aplustridae | *Parvaplustrum sp.* | 0 | 0 | 0 | 0 | 1 |
| Mollusca | Cataegidae | *Kanoia myronfeinbergi* | 0 | 0 | 0 | 0 | 1 |
| Mollusca | Lepetodrilidae | *Gorgoleptis emarginatus* | 1 | 0 | 0 | 0 | 0 |
| Mollusca | Lepetodrilidae | *Lepetodrilus cristatus* | 1 | **1** | 0 | 0 | 0 |
| Mollusca | Lepetodrilidae | *Lepetodrilus elevatus* | 1 | **1** | 0 | 0 | 0 |
| Mollusca | Lepetodrilidae | *Lepetodrilus guaymasensis* | 0 | 0 | 0 | 0 | 1 |
| Mollusca | Lepetodrilidae | *Lepetodrilus ovalis* | 1 | 0 | 0 | 0 | 0 |
| Mollusca | Lepetodrilidae | *Lepetodrilus pustulosus* | 1 | 0 | 0 | 0 | 0 |
| Mollusca | Neolepetopsidae | *Euleptopsis vitrea* | 1 | **1** | 0 | 0 | 0 |
| Mollusca | Neolepetopsidae | *Neolepetopsis gordensis* | 1 | 0 | 0 | 1 | 0 |
| Mollusca | Neolepetopsidae | *Neolepetopsis occulta* | 1 | 0 | 0 | 0 | 0 |

1 = present
**bold** is range extension
* is 1st sighting in the GoC
0 = absent

| | | | | | | | |
|---|---|---|---|---|---|---|---|
| Mollusca | Neolepetopsidae | *Neolepetopsis verruca* | 1 | 0 | 0 | 0 | 0 |
| Mollusca | Neolepetopsidae | *Paralepetopsis sp.* | 0 | 0 | 1 | 1 | 0 |
| Mollusca | Neomphalidae | *Cyathermia naticoides* | 1 | 0 | 0 | 0 | 0 |
| Mollusca | Neomphalidae | *Melanodrymia aurantiaca* | 0 | 1 | 0 | 0 | 0 |
| Mollusca | Neomphalidae | *Neomphalus fretterae* | 1 | 0 | 0 | 0 | 0 |
| Mollusca | Neomphalidae | *Pachydermia laevis* | 1 | 0 | 0 | 0 | 0 |
| Mollusca | Neomphalidae | *Planorbidella planispira* | 1 | 0 | 0 | 0 | 0 |
| Mollusca | Neomphalidae | *Retiskenea diploura* | 0 | 0 | 0 | 0 | 1 |
| Mollusca | Nuculanidae | *Nuculana grasslei* | 0 | 0 | 0 | 0 | 1 |
| Mollusca | Peltospiridae | *Echinopelta fistulosa* | 1 | 0 | 0 | 0 | 0 |
| Mollusca | Peltospiridae | *Hirtopelta hirta* | 1 | 0 | 0 | 0 | 0 |
| Mollusca | Peltospiridae | *Hirtopelta tufari* | 1 | 0 | 0 | 0 | 0 |
| Mollusca | Peltospiridae | *Lirapex granularis* | 1 | 0 | 0 | 0 | 0 |
| Mollusca | Peltospiridae | *Nodopelta sp.* | 1 | 1 | 0 | 0 | 0 |
| Mollusca | Peltospiridae | *Peltospira delicata* | 0 | 0 | 1* | 0 | 0 |
| Mollusca | Peltospiridae | *Peltospira n. sp.* | 0 | 1 | 0 | 0 | 0 |
| Mollusca | Peltospiridae | *Peltospira operculata* | 1 | 0 | 0 | 0 | 0 |
| Mollusca | Peltospiridae | *Rhynchopelta concentrica* | 1 | 0 | 0 | 0 | 0 |
| Mollusca | Provannidae | *Provanna ios* | 1 | 0 | 1* | 1 | 0 |
| Mollusca | Provannidae | *Provanna laevis* | 0 | 0 | 1 | 0 | 1 |
| Mollusca | Provannidae | *Provanna lomana* | 0 | 0 | 0 | 0 | 1 |
| Mollusca | Provannidae | *Provanna muricata* | 1 | 0 | 0 | 0 | 0 |
| Mollusca | Provannidae | *Provanna n. sp 2* | 0 | 0 | 1 | 0 | 0 |
| Mollusca | Pseudococculinidae | *Amphiplica sp.* | 1 | 0 | 0 | 0 | 0 |
| Mollusca | Pyramidellidae | *Eulimella lomana* | 1 | 0 | 0 | 1 | 0 |
| Mollusca | Pyropeltidae | *Pyropelta corymba* | 0 | 0 | 0 | 0 | 1 |
| Mollusca | Pyropeltidae | *Pyropelta musaica* | 0 | 0 | 0 | 0 | 1 |
| Mollusca | Solemyidae | *Acharax aff johnsoni* | 0 | 0 | 0 | 0 | 1 |
| Mollusca | Trochidae | *Bathymargarites aff. symplector* | 1 | **1** | 0 | 0 | 0 |
| Mollusca | Turbinidae | Turbinidae | 0 | 0 | 1 | 1 | 0 |
| Mollusca | Vesicomyidae | *'Calyptogena' magnifica* | 1 | **1** | 0 | 0 | 0 |
| Mollusca | Vesicomyidae | *Calyptogena lepta* | 0 | 0 | 0 | 0 | 1 |
| Mollusca | Vesicomyidae | *Calyptogena costaricana* | 0 | 0 | 0 | 1 | 0 |
| Mollusca | Vesicomyidae | *Archivesica gigas* | 0 | 0 | 0 | 0 | 1 |
| Mollusca | Vesicomyidae | *Archivesica sp 7* | 0 | 0 | 1* | 1 | 0 |
| Mollusca | Vesicomyidae | *Archivesica* mtV | 0 | 0 | 0 | 1 | 0 |
| Mollusca | Vesicomyidae | *Phreagena soyoae* | 0 | 0 | 0 | 0 | 1 |
| Porifera | | *Caulophacus cyanae* | 0 | 1 | 0 | 0 | 0 |
| Xenoturbella | Xenoturbellidae | *Xenoturbella profunda* | 0 | 0 | 1* | 0 | 0 |

**Table S2.** PCR primers and conditions used to amplify and sequence genes for identification for each taxonomic group extracted.

| Primers | Ref | Reaction Condition | Fragment | Primer Sequence 5'->3' | Group |
|---|---|---|---|---|---|
| LSU26F/<br>LSU657R | [1] | 1 | 28S | LSU26F:<br>AATGATACGGCGACCACCGAGATCTACACTATGGTAATTGTACCCGCTGAACTTAAGCATAT<br>LSU657R:<br>CAAGCAGAAGACGGCATACGAGATGTCGAATTTGCGAGTCAGTCAGCCCTTGGTCCGTGTTTCAAGAC | Illumina MiSeq HTS |
| mlCOIintF | [2] | 1 | COI | mlCOIintF:<br>AATGATACGGCGACCACCGAGATCTACACTATGGTAATTGTGGWACWGGWTGAACWGTWTAYCCYCC | Illumina MiSeq HTS |
| jgHCO2198 | [3] | 1 | COI | jgHCO2198:<br>CAAGCAGAAGACGGCATACGAGATCACTGGTGCATAAGTCAGTCAGCCTAAACTTCAGGGTGACCAAAAAATCA | Illumina MiSeq HTS |
| LCO1490/<br>HCO2198 | [4] | 2 | COI | LCO1490: GGTCAACAAATCATAAAGATATTGG<br>HCO2198: TAAACTTCAGGGTGACCAAAAAATCA | Gastropods |
| jgLCO1490/<br>HCO2198 | [3] | 2 | COI | jgLCO1490:<br>TITCIACIAAYCAYAARGAYATTGG<br>HCO2198: TAAACTTCAGGGTGACCAAAAAATCA | Provannids |
| COIF/COIR | [5] | 2 | COI | COIF:<br>TCMACTAATCAYAARGAYATTGGNA<br>COIR:<br>CCDCTTAGWCCTARRAARTGTTGNGG | Annelids, Lepetodrilids |
| VesLCO/<br>VesHCO | [6] | 2 | COI | VesLCO:<br>TTAATAGGAACTGCTTTTAG<br>VesHCO:<br>TCACCCAAACCAgCAggATC | Vesicomyids |
| galaCOIF/<br>galaCOIR | [7] | 2 | COI | galaCOIF:<br>CATCACTWAGWTTRATYA TTCGAGCAGAA<br>galaCOIR:<br>GAAYAGGRTCTCCTC CTC CTAC | Arthropods |
| AnthCOIIIF/<br>AnthCOIIIR | [8] | 2 | COIII | AnthCOIIIF: ACTTTTCAAGGTCTTCACACCRTGGTT<br>AnthCOIIIR: CAAACCACATCTACAAAATGCCAATATC | Actinarians |
| Anem16SA/<br>Anem16SB | [8] | 2 | 16S rRNA | Anem16SA:<br>CACTGACCGTGATAATGTAGCGT<br>Anem16SB:<br>CCCCATGGTAGCTTTTATTCG | Actinarians |
| 27F/1492R | [9] | 3 | 16S | 27F:<br>AGAGTTTGATCMTGGCTCAG<br>1492R:<br>TACGGYTACCTTGTTACGACTT | Bacteria, Archaea |
| symITS16SF/<br>sym23SR | [10] | 3 | ITS | SymITS16SF:<br>GAAGTCGTAACAAGG<br>Sym23SR:<br>CAAGGCATCCACCGT | Bacteria |

PCR primer conditions:

1) Touchdown protocol reactions in triplicate: 95°C-10', (94°C-10S, 62°C-30S, 68°C-1')×16 -1°C each cycle), (94°C-10S, 46°C-30S, 68°C-1')×25, 72°C-10', 4°C hold.

2) 95°C-10', (95°C-1', 50°C-1', 72°C-1')×35, 72°C-7', 4°C hold.

3) 95°C-10', (92°C-1', 60°C-30S, 68°C-1')×25, 72°C-6', 4°C hold.

**Supplemental Table 3.** Larval detection, via environmental DNA analysis from 100-µm filtered seawater collected at 5 different depths at each site. Taxa detected in the water column are indicated via an *l.* Taxa collected or observed via video are indicated by an x.

| **Animal taxa** | **AR Vents** | **PB Vents** | **PTF Seeps** |
|---|---|---|---|
| **Annelida - Polychaeta** | | | |
| Siboglinidae | | | |
| *Riftia pachyptila* | x, *l* | x | *l* |
| *Oasisia* haplotype IIA | x, *l* | | |
| Alvinellidae | | | |
| *Alvinella pompejana* | x , *l* | | |
| Spionidae | | | |
| *Prionospio sandersi* | x , *l* | | |
| Maldanidae | | *l* | |
| *Nicomache* cf. *arwidssoni* | x | | |
| *Nicomache venticola* | | | |
| **Mollusca - Bivalvia** | | | |
| Vesicomyidae | | | |
| *Calyptogena costaricana* | *l* | *l* | x, *l* |
| *"Calyptogena" magnifica* | x, *l* | | *l* |
| **Mollusca - Gastropoda** | | | |
| Lepetodrilidae | | | |
| *Lepetodrilus elevatus* | x, *l* | | *l* |
| Provannidae | | | |
| *Provanna* n sp 2 | *l* | x | |
| Melanodrymidae | | | |
| *Melanodrymia aurantiaca* | x, *l* | | *l* |
| **Cnidaria** | *l* | *l* | *l* |
| Small white actinarian | x | x | |
| White actinarian | | x | |
| Red actinarian | | x | |
| **Crustacea - Decapoda** | | | |
| *Lebbeus (carinatus)* | x | | *l* |

AR = Alarcón Rise, PB = Pescadero Basin,
PTF = Pescadero Transform Fault, GUY = Guaymas Basin

**Supp Table 4:** Faunal type and abundance (#/$m^2$) for Pescadero Basin vents, identified from 10 video transects at four venting structures.

| Taxa | **Mater horn** | **C vent I** | **C vent II** | **Z vent I** | **Z vent II** | **Z vent III** | **Z vent IV** | **P vent I** | **P vent II** | **P vent III** |
|---|---|---|---|---|---|---|---|---|---|---|
| **Cnidaria** | | | | | | | | | | |
| Actiniaria sp. 1 | 20.03 | 14.73 | 29.24 | 67.93 | 8.82 | 4.30 | 14.42 | 24.85 | 30.14 | 17.42 |
| Actiniaria sp. 2 | - | - | - | - | - | - | - | - | - | - |
| Actiniaria-other | 2.29 | - | - | - | 0.14 | 0.13 | - | - | - | 0.07 |
| Zoantharia | 6.86 | 0.07 | - | 21.76 | - | 1.27 | - | - | - | - |
| Cerianthidae | - | 0.37 | - | 4.52 | - | - | 6.81 | - | 0.60 | 0.73 |
| **Annelida** | | | | | | | | | | |
| Alvinellidae | - | - | - | 0.26 | - | - | - | 0.42 | - | - |
| Amphinomidae | - | 0.15 | 0.08 | 0.68 | - | - | - | - | - | - |
| *Hesiolyra bergi* | - | - | - | - | - | - | - | - | - | - |
| *Nereis cf. sandersi* | - | - | - | - | - | - | - | - | - | - |
| *Oasisia* aff *alvinae* | 57.18 | 318.88 | - | 30.98 | 11.20 | 783.54 | 0.53 | - | 2422.97 | 451.38 |
| *Peinaleopolynoe* sp. 1 | 32.02 | 16.45 | 11.5 | 14.09 | 24.79 | 7.72 | 10 | 12.12 | 22.25 | 4.8 |
| *Riftia pachyptila* | - | - | - | - | - | - | - | - | - | - |
| *Protis* n sp. | - | - | - | - | - | - | - | - | - | - |
| *Laminatubus* spp. | - | - | - | - | - | - | - | - | - | - |
| Serpulidae-other | - | 0.07 | - | - | - | - | - | - | - | - |
| **Mollusca** | | | | | | | | | | |
| *C. magnifica* | - | - | - | - | - | - | - | - | - | - |
| Gastropoda | - | 0.07 | - | 0.51 | 0.42 | - | - | - | 0.12 | - |
| Patellogastropoda | - | - | - | - | - | - | - | - | - | - |
| Vesicomyidae | - | - | - | - | - | - | - | - | 0.12 | - |
| **Crustacea** | | | | | | | | | | |
| Amphipoda | - | - | - | 0.26 | - | - | - | - | - | - |
| *B. thermydron* | - | - | - | - | - | - | - | - | - | - |
| *L. carinatus* | - | 0.07 | - | - | - | - | - | - | - | - |
| Galatheidae | - | 0.15 | - | - | 0.28 | - | - | 0.14 | 0.12 | 0.15 |
| **Vertebrata** | | | | | | | | | | |
| *T. cerberus* | - | - | 0.08 | 0.09 | 0.28 | 0.38 | - | - | - | - |
| Transect width (m) | 1.06 | 1.07 | 0.78 | 0.63 | 1.4 | 1 | 1.19 | 1.08 | 0.95 | 1.06 |
| Transect length (m) | 3.3 | 12.5 | 16.4 | 18.6 | 5.1 | 7.9 | 9.5 | 13.3 | 8.8 | 13 |
| Transect area (m^2) | 3.50 | 13.38 | 12.79 | 11.72 | 7.14 | 7.9 | 11.31 | 14.36 | 8.36 | 13.78 |
| Dive # | D751 | D751 | D751 | D750 | D757 | D757 | D757 | D751 | D757 | D757 |
| Transect # | T1 | T2 | T3 | T1 | T1 | T2 | T3 | T4 | T4 | T5 |

**Supp Table 5:** Faunal type and abundance (#/$m^2$) for Alarcón Rise vents, identified from 14 video transects at three vent fields.

| Taxa | **Meyibo I** | **Meyibo II** | **Meyibo III** | **Meyibo IV** | **Tzab-ek I** | **Tzab-ek II** | **Tzab-ek III** | **Tzab-ek IV** | **Tzab-ek V** | **Ja-Sit I** | **Ja-Sit II** | **Ja-Sit III** | **Ja-Sit IV** | **Ja-Sit V** |
|---|---|---|---|---|---|---|---|---|---|---|---|---|---|---|
| **Cnidaria** | | | | | | | | | | | | | | |
| Actiniaria sp. 1 | - | - | - | 0.83 | - | 1.50 | - | - | - | 1.99 | 0.12 | 1.44 | - | 0.11 |
| Actiniaria sp. 2 | - | - | - | - | - | - | - | - | - | - | - | - | 131.36 | 0.97 |
| Actiniaria-other | - | - | - | 0.28 | - | - | - | - | - | - | - | - | 0.26 | - |
| Zoantharia | - | - | - | - | - | - | - | - | - | - | - | - | - | - |
| Cerianthidae | - | - | - | - | - | - | - | - | - | - | - | - | 0.13 | - |
| **Annelida** | | | | | | | | | | | | | | |
| Alvinellidae | 40.00 | 4.08 | - | 5.28 | - | 39.47 | - | - | - | 0.66 | - | - | - | 0.05 |
| Amphinomidae | - | - | - | - | - | - | - | - | - | - | - | - | - | - |
| *Hesiolyra bergi* | 0.78 | - | - | - | - | - | - | 0.06 | - | 0.66 | - | 0.08 | - | - |
| *Nereis cf. sandersi* | - | 0.51 | - | - | 0.41 | 1.13 | - | 0.25 | - | 0.66 | - | 0.23 | - | 0.04 |
| *Oasisia* aff *alvinae* | - | - | - | 12.22 | - | 200.00 | 191.06 | 0.86 | - | - | - | - | - | - |
| *Peinaleopolynoe* sp. 1 | - | - | - | 0.28 | 0.35 | 0.38 | - | - | - | - | 0.06 | 0.23 | - | - |
| *Riftia pachyptila* | 504.31 | 730.10 | 474.77 | 102.78 | - | 38.72 | - | - | 16.13 | 66.98 | 12.46 | 1.98 | - | - |
| *Protis* n. sp. | 370.98 | 125.00 | 34.02 | 380.28 | - | - | 126.99 | 30.89 | - | - | - | 0.08 | - | 0.02 |
| *Laminatubus* spp. | 2.35 | 6.12 | - | 7.78 | 0.14 | - | 41.19 | 11.70 | 52.02 | 18.57 | 9.44 | - | 0.13 | - |
| Serpulidae-other | - | - | - | - | - | - | 6.69 | - | - | - | - | 15.19 | - | 1.99 |
| **Mollusca** | | | | | | | | | | | | | | |
| *C. magnifica* | - | - | - | 0.56 | - | - | - | - | - | - | - | - | - | - |
| Gastropoda | - | - | - | 277.50 | - | 142.86 | 0.06 | - | - | - | - | - | - | - |
| Patellogastropoda | 1020.39 | 1224.49 | 3822.63 | 2527.78 | 7.81 | 3460.15 | 231.11 | - | 120.97 | 72.94 | 5.93 | - | - | 4.02 |
| Vesicomyidae | - | - | - | - | - | - | 0.06 | - | - | - | - | - | - | 0.02 |
| **Crustacea** | | | | | | | | | | | | | | |
| Amphipoda | - | - | - | - | 10.37 | 409.40 | - | - | - | - | - | - | - | - |
| *B. thermydron* | 10.98 | 11.22 | 8.03 | 1.11 | 0.07 | 1.50 | 0.23 | 0.18 | 0.81 | 3.32 | 0.65 | 2.20 | 0.39 | 0.18 |
| *L. carinatus* | - | - | - | 0.28 | - | 1.50 | 0.23 | - | - | - | 0.06 | 0.08 | - | - |
| Galatheidae | - | 1.02 | 6.88 | 0.56 | 0.14 | 3.76 | 1.94 | 0.18 | 0.40 | 7.96 | 0.36 | 0.46 | 0.39 | 0.05 |
| **Vertebrata** | | | | | | | | | | | | | | |
| *T. cerberus* | 25.88 | 61.22 | 35.17 | 1.11 | 0.07 | 1.50 | 0.17 | 0.80 | 0.81 | 1.99 | 0.30 | 1.75 | - | 0.15 |
| Transect width (m) | 0.85 | 1.09 | 1.09 | 1.2 | 1.00 | 0.95 | 1.37 | 1.15 | 1.24 | 1.16 | 1.25 | 1.2 | 1.17 | 1.8 |
| Transect length (m) | 1.5 | 1.8 | 2.4 | 3 | 14.5 | 2.8 | 12.76 | 14.19 | 2 | 1.3 | 13.48 | 10.97 | 6.5 | 30.4 |
| Transect area (m^2) | 1.28 | 1.96 | 2.62 | 3.6 | 14.47 | 2.66 | 17.48 | 16.32 | 2.48 | 1.51 | 16.85 | 13.16 | 7.61 | 54.72 |
| Dive # | D752 | D752 | D752 | D752 | D753 | D753 | D753 | D753 | D753 | D754 | D754 | D754 | D754 | D754 |
| Transect # | T1 | T2 | T3 | T4 | T1 | T2 | T3 | T4 | T5 | T1 | T2 | T3 | T4 | T5 |

**Supp Table 6.** Mean (±SD) and individual values of $\delta^{13}C$ and $\delta^{15}N$ of the fauna from Pescadero Basin vents (PB), Alarcón Rise vents (AR), Pescadero Transform Fault seeps (PTF), as well as non vent/seep fauna. N = number of individuals.

| # in figurePB vent Taxa | Trophic guild | N | $\delta^{13}C$‰ | $\delta^{15}N$‰ |
|---|---|---|---|---|
| **Cnidaria** | | | | |
| Actiniaria indiv. 1 | suspension-feeder | 1 | -35.6 | 2.7 |
| Actiniaria indiv. 2 | suspension-feeder | 1 | -32.6 | 6.8 |
| Actiniaria indiv. 3 | suspension-feeder | 1 | -33.8 | 2.1 |
| Actiniaria indiv. 4 | suspension-feeder | 1 | -37.9 | 1.0 |
| Actiniaria indiv. 5 | suspension-feeder | 1 | -19.6 | 5.6 |
| Actiniaria group 6 | suspension-feeder | 4 [a] | -23.6 | 4.5 |
| Actiniaria group 7 | suspension-feeder | 26 [a] | -20.8 | 14 |
| **Annelida** | | | | |
| Amphinomidae sp. | predator | 1 | -17.9 | 6.7 |
| Amphinomidae sp.2 | predator | 1 | -22.3 | 4.5 |
| *Oasisia* aff *alvinae* | symbiont-bearing | 6 | -14.7 (± 1.4) | 1.9 (± 0.4) |
| *Riftia pachyptila* | symbiont-bearing | 1 | -13.1 | 3.8 |
| *Paralvinella* sp. | bacterivorous | 2 [a] | -20.5 | -0.8 |
| **Mollusca** | | | | |
| *Archivesica* sp. 7 | symbiont-bearing | 1 | -35.3 | -2.3 |
| **Crustacea** | | | | |
| *Munidopsis scotti* | predator/scavenger | 1 | -19.2 | 10.5 |
| **AR vent Taxa** | | | | |
| **Porifera** | | | | |
| *Caulophacus cyanae* | suspension-feeder | 3 | -26.7 (± 1.6) | 17.6 (± 0.4) |
| **Cnidaria** | | | | |
| Actiniaria sp. | suspension-feeder | 2 | -12.1 (± 2.1) | 8.9 (± 0.1) |
| **Annelida** | | | | |
| *Nereis* sp. | predator | 1 | -17.1 | 4.0 |
| *Alvinella pompejana* | symbiont-bearing | 2 | -9.4 (± 2.0) | 0.9 (± 0.1) |
| *Riftia pachyptila* | symbiont-bearing | 5 | -11.1 (± 1.7) | 4.1 (± 0.3) |
| **Mollusca** | | | | |
| *Calyptogena magnifica* | symbiont-bearing | 3 | -34.2 (± 1.4) | -4.6 (± 1.0) |
| **Crustacea** | | | | |
| *Bythograea thermydron* | predator | 1 | -11.7 | 8.2 |
| *Lebbeus* sp. | predator | 1 | -14.8 | 8.2 |
| **Vertebrata** | | | | |
| *Thermarces cerberus* | predator | 1 | -12.0 | 9.9 |
| **PTF seep Taxa** | | | | |

| | | | | |
|---|---|---|---|---|
| **Annelida** | | | | |
| *Escarpia spicata* | symbiont-bearing | 2 | -17.7 (± 0) | 0.3 (± 0.2) |
| *Lamellibrachia barhami* | symbiont-bearing | 2 | -17.0 (± 1.1) | -1.2 (± 1.5) |
| **Mollusca** | | | | |
| *Calyptogena costaricana* | symbiont-bearing | 1 | -39.5 | -9.1 |
| **Non vent/seep Taxa (site)** | | | | |
| **Cnidaria** | | | | |
| *Actinaria* sp. 8 (PB) | suspension-feeder | 2 | -18.9 (± 0.4) | 18.7 (± 0.5) |
| **Echiura** | | | | |
| Echiuridae sp. (AR) | detritivore | 2 [a] | -21.9 | 7.2 |
| **Echinodermata** | | | | |
| *Holothuroidea* sp. (AR) | detritivore | 1 | -19.0 | 17.2 |

[a] = small specimens which were pooled prior to analysis

## Supplemental References


[1] Park, C.H., Kim, K.M., Elvebakk, A., Kim, O.S., Jeong, G. & Hong, S.G. 2015 Algal and fungal diversity in Antarctic lichens. *The Journal of eukaryotic microbiology* **62**, 196–205. (doi:10.1111/jeu.12159).

[2] Leray, M., Yang, J.Y., Meyer, C.P., Mills, S.C., Agudelo, N., Ranwez, V., Boehm, J.T. & Machida, R.J. 2013 A new versatile primer set targeting a short fragment of the mitochondrial COI region for metabarcoding metazoan diversity: application for characterizing coral reef fish gut contents. *Frontiers in Zoology* **10**, 1–14. (doi:10.1186/1742-9994-10-34).

[3] Geller, J.B., Meyer, C.P., Parker, M. & Hawk, H. 2013 Redesign of PCR primers for mitochondrial cytochrome c oxidase subunit I for marine invertebrates and application in all-taxa biotic surveys. *Molecular Ecology Resources* **13**, 851-861.

[4] Folmer, O., Black, M., Hoeh, W., Lutz, R. & Vrijenhoek, R. 1994 DNA primers for amplification of mitochondrial cytochrome C oxidase subunit I from metazoan invertebrates. *Molecular Marine Biology and Biotechnology* **3**, 294-299.

[5] Nelson, K. & Fisher, C. 2000 Absence of cospeciation in deep-sea vestimentiferan tube worms and their bacterial endosymbionts. *Symbiosis* **28**, 1-15.

[6] Peek, A., Gustafson, R., Lutz, R. & Vrijenhoek, R. 1997 Evolutionary relationships of deep-sea hydrothermal vent and cold-water seep clams (Bivalvia: Vesicomyidae): Results from the mitochondrial cytochrome oxidase subunit I. *Marine Biology* **130**, 151-161.

[7] Jones, W.J. & Macpherson, E. 2007 Molecular phylogeny of the East pacific Rise squat lobsters of the genus *Munidopsis* (Decapoda: Galitheidae) with the descriptions of seven new species. *Journal of Crustacean Biology* **27**, 477-501.

[8] Geller, J.B. & Walton, E.D. 2001 Breaking up and getting together: Evolution of symbiosis and cloning by fission in sea anemones (Genus *Anthopleura*). *Evolution* **55**, 1781-1794.

[9] Lane, D.J., Weisberg, W.G., Barns, S.M. & Pelletier, D.A. 1991 16S ribosomal DNA amplification for phylogenetic study. *Journal of Bacteriology* **173**, 697–703.

[10] Won, Y.-J., Hallam, S.J., O'Mullan, G.D., Pan, I.L., Buck, K.R. & Vrijenhoek, R.C. 2003 Environmental acquisition of thiotrophic endosymbionts by deep-sea mussels of the genus *Bathymodiolus*. *Applied and Environmental Microbiology* **69**, 6785-6792.
